# Jahresberichte 2022 aus den GfH-Kommissionen und GfH-Arbeitskreisen

**DOI:** 10.1515/medgen-2023-2013

**Published:** 2023-04-05

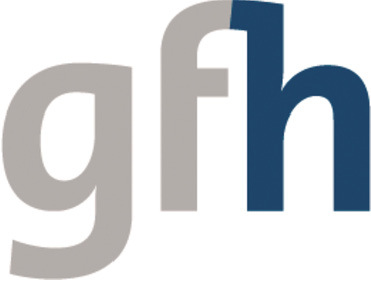



## Jahresbericht 2022 der Kommission für Grundpositionen und ethische Fragen der GfH

Prof. Dr. med. Christian Netzer, Köln (Sprecher)

Prof. Dr. med. Stefan Aretz, Bonn (stellv. Sprecher)

Dr. med. Martin Kehrer, Tübingen

Prof. Dr. med. Uwe Kornak, Göttingen

Dr. med. Felicitas Maier, München

Dr. med. Rixa Woitschach, Hamburg

Prof. Dr. med. Dr. rer. nat. Birgit Zirn, Stuttgart

Die Kommission hat im Jahr 2022 drei Videokonferenzen abgehalten. Die beiden thematischen „Großbaustellen“ der Kommissionsarbeit der letzten Jahre – die Neuauflage der Stellungnahme zum Umgang mit Zusatz- und Zufallsbefunden bei Exom- und Genomanalysen sowie die Stellungnahme zur genetischen Pränataldiagnostik bei auffälligen Ultraschallbefunden – konnten abgeschlossen und dem Vorstand der GfH übergeben werden. Eckpunkte der Stellungnahme zum Umgang mit Zusatz- und Zufallsbefunden wurden vom Sprecher der Kommission auch auf der BVDH-Herbsttagung in Köln vorgestellt, und Anregungen aus der dortigen regen Diskussion sind in den finalen Entwurf eingeflossen. Beide Stellungnahmen sind aufeinander abgestimmt und mit der neuen „Richtlinie der Gendiagnostik-Kommission (GEKO) für die Anforderungen an die Inhalte der Aufklärung bei genetischen Untersuchungen zu medizinischen Zwecken“ harmonisiert. Die Kommission wird versuchen, die im Februar 2023 eingegangenen Kommentare des GfH-Vorstandes rasch zu bearbeiten, so dass die Stellungnahmen voraussichtlich im Frühjahr 2023 veröffentlicht werden können.

Für das Jahr 2023 hat sich die Kommission vorgenommen zu diskutieren, welche Möglichkeiten es gibt, mehr Patient:innen einen Zugang zur stark nachgefragten humangenetischen Expertise zu verschaffen. Neben dem Modell des „Genetic counsellors“, das für Entlastung von Fachärztinnen und Fachärzten für Humangenetik sorgen könnte, soll auch eruiert werden, ob sich das Fach durch die hohen Anforderungen der S2k-Leitlinie „Humangenetische Diagnostik und Beratung“ an die schriftliche Dokumentation der genetischen Beratung (Stichwort „Beratungsbrief“) nicht selbst einen „Mühlstein“ um den Hals gelegt hat, der auch aus ethischer Perspektive unter den aktuellen Bedingungen der Ressourcenknappheit zumindest bei bestimmten Fragestellungen durchaus kritisiert werden könnte.

Einige Mitglieder arbeiten schon seit vielen Jahren in der Kommission. Insofern kam die Anfrage der „Jungen Humangenetik“ (JH), ob Frau Dr. Johanna Tecklenburg als Vertreterin der JH in der Kommission mitarbeiten könne, zur rechten Zeit. Frau Dr. Tecklenburg wird sich auf der GfH-Tagung in Kassel zur Wahl stellen. Birgit Zirn wird nach neun Jahren wertvoller Arbeit aus der Kommission ausscheiden.

Bericht: Christian Netzer

## Jahresbericht 2022 der Fachhumangenetiker-Kommission der GfH

Prof. Dr. biol. hum. Ulrich Zechner, Köln (Sprecher)

Dr. rer. nat. Sönke Arps, Hamburg

Dr. med. Bernd Auber, Hannover

Dr. rer. nat. Birgitta Gläser, Freiburg

Dr. rer. nat. Katrin Hinderhofer, Heidelberg

Dr. rer. nat. Christine Neuhaus, Köln

### Fachhumangenetiker-Weiterbildung

Momentan werden insgesamt 234 FachhumangenetikerInnen (GfH) in der Statistik der GfH-Geschäftsstelle geführt.

**Abbildung 1:**
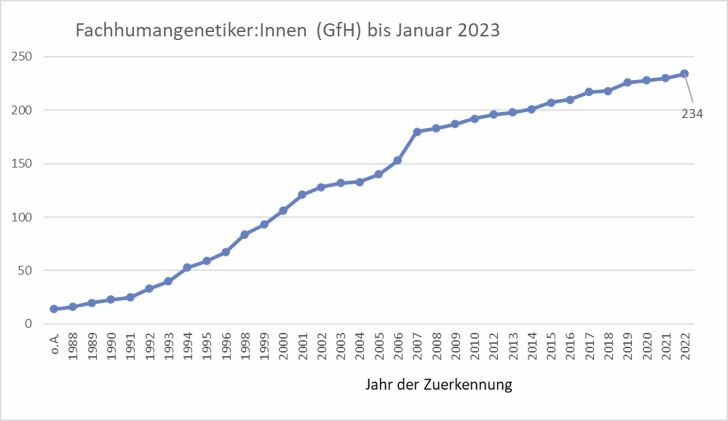
Zahl der Zuerkennungen bis Januar 2023

Das Interesse an der Weiterbildung ist aktuell weiterhin sehr hoch. Im Berichtszeitraum haben 38 NaturwissenschaftlerInnen den Beginn ihrer Weiterbildung zur/zum Fachhumangenetiker/in (GfH) angemeldet; da ein Beginn der Weiterbildung ab Anmeldung Monate rückwirkend zuerkannt werden kann, haben im Jahr 2022 insgesamt 21 und im Jahr 2021 rückwirkend bereits zehn NaturwissenschaftlerInnen ihre Weiterbildung begonnen (Abb. 2).

Gegenwärtig befinden sich 310 Personen in der Weiterbildung zur/zum Fachhumangenetiker/In. 

Im Berichtszeitraum haben vier FachhumangenetikerInnen (GfH) ihre Weiterbildung erfolgreich mit dem Fachgespräch abgeschlossen.

**Abbildung 2:**
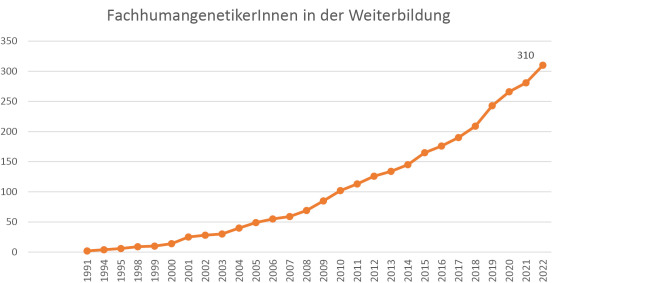
WeiterbildungskandidatInnen bis einschließlich 31.12.2022

### Erteilung von Weiterbildungsbefugnissen

Im Berichtszeitraum wurden zwölf neue Befugnisse zur Weiterbildung erteilt.

Im ersten Halbjahr 2023 werden alle Weiterbildungsbefugten (inkl. der vor Inkrafttreten der novellierten Weiterbildungsordnung (WBO) qua Amt weiterbildungsbefugten LeiterInnen universitärer humangenetischer Einrichtungen) von der Geschäftsstelle der GfH angeschrieben und gebeten, im Rahmen einer Online-Umfrage Daten zur Prüfung der vorliegenden Weiterbildungsbefugnisse und Aktualisierung der auf der GfH-Website verfügbaren Liste der Weiterbildungsbefugten zur Verfügung zu stellen.

### Änderungen der Weiterbildungsordnung und Geschäftsordnung

Mit einstimmigem Beschluss des Vorstands und der Kommission wurden folgende Änderungen im Abschnitt B (Gegenstandskatalog) der WBO vom 07.05.2021 durchgeführt:

Ergänzung auf Seite 7, Absatz 4:
  „In begründeten Ausnahmefällen ist auch die Aufnahme von Untersuchungen, die retrospektiv ausgewertet und mit einem schriftlichen Befundbericht abgeschlossen werden, zulässig.“
Änderung bzw. Ergänzung (jeweils fett und unterstrichen) auf Seite 10 unten unter „Fortbildungen“:
  mindestens **1,5**-tägiger Kurs der Akademie Humangenetik **oder des European Board of Medical Genetics** oder Gastaufenthalte in einer Weiterbildungsstätte gemäß § 5 und § 6 (Abschnitt A)

Mit einstimmigem Beschluss des Vorstands und der Kommission wurde entschieden, dass die in der Geschäftsordnung festgelegte Anzahl der Kommissionsmitglieder von sechs auf sieben Personen erhöht wird. Neben den bisherigen sechs Kommissionsmitgliedern mit abgeschlossener humangenetischer Weiterbildung fungiert als siebtes Kommissionsmitglied eine Person, die gleichzeitig Mitglied der Jungen Humangenetik ist und ihre Weiterbildung zum Fachhumangenetiker/zur Fachhumangenetikerin (GfH) begonnen oder abgeschlossen haben muss. Dieses Mitglied ist nur nach bereits erfolgtem Abschluss der Weiterbildung befugt, Anmeldungen zur Weiterbildung zu bearbeiten, Anträge auf Zulassung zum Fachgespräch sowie Anträge auf Berechtigung zur Weiterbildung von FachhumangenetikerInnen (GfH) zu begutachten und als Gutachter bei Fachgesprächen zu fungieren.

### Fortbildungsangebote für FachhumangenetikerInnen

Die Kommission hat in Abstimmung mit der Akademie Humangenetik und den beteiligten ReferentInnen Konzepte für drei Kurse erarbeitet, die gemäß der novellierten WBO fakultativ als Ersatz für jeweils 15 Laboruntersuchungen aus den Kategorien C1/C4 (postnatale Zytogenetik und FISH-Analysen), C2 (Tumorzytogenetik) sowie C3 (pränatale Zytogenetik) der WBO dienen können. Die beiden Kurse für die Kategorien C2 (09.–10.03.2023) bzw. C1/C4 (24.–25.03.2023) sind bereits auf der Webseite der Akademie (www.akademie-humangenetik.de) angekündigt und zur Registrierung freigeschaltet. Ein weiterer Kurs, der voraussichtlich als Ersatz für Laboruntersuchungen aus der Kategorie M10 (epigenetische Analysen) dienen kann, ist in Planung. Die Kommission verweist zudem auf das weitere Kursangebot der Akademie Humangenetik, das die Weiterbildung zum/zur Fachhumangenetiker/in (GfH) begleitet und unterstützt.

### Europäische Anerkennung

Das Zertifikat „European registered Clinical Laboratory Geneticist (ErCLG)“ wurde in der aktuellen Antragsrunde 2022/2023 von acht Antragstellern aus Deutschland (davon zwei FachhumangenetikerInnen (GfH), sowie zwei Antragsteller mit nationalen CLG-Zertifikaten anderer Länder) beantragt und wird nach Prüfung durch das European Board of Medical Genetics (EBMG), Abteilung „Clinical Laboratory Geneticists“ im Jahr 2023 in voraussichtlich sechs Fällen bewilligt werden.

Beide FachhumangenetikerInnen (GfH) hatten das ErCLG-Zertifikat bereits zuvor erworben und mussten in der aktuellen Runde lediglich eine Zertifikatsverlängerung beantragen. Leider reichten vier weitere FachhumangenetikerInnen (GfH) keinen Antrag zur Rezertifizierung ein. Diese Personen können die Rezertifizierung auf Wunsch gerne in der kommenden Antragsrunde nachholen.

Die Zahl der gültigen ErCLG-Zertifikate beträgt europaweit aktuell 416 (Stand Januar 2023), darunter aber 23 Zertifikate, die am 30.04.2023 auslaufen und für die keine Verlängerung beantragt wurde. Zudem ist die Bewertung von 35 Anträgen, die über den sogenannten „Gruppe 3-Weg“ gestellt wurden, noch nicht abgeschlossen; daher ist davon auszugehen, dass die Zahl der ErCLG-ZertifikatsträgerInnen Ende 2023 weiterhin über 410 betragen wird. Die Kommission empfiehlt daher, die Zertifizierung als ErCLG aufrecht zu erhalten.

Der wiederholte Dank der Kommission gilt dem EBMG für seine erfolgreiche Arbeit bei der Entwicklung und Implementierung von europäischen Ausbildungsstandards in der Humangenetik sowie der Profilierung des Berufsbildes „Fachhumangenetiker/in GfH/ErCLG“.

Abschließend danke ich allen Kommissionsmitgliedern sowie den KursreferentInnen, dem Vorstand und der Geschäftsstelle der GfH für die hervorragende und verlässliche Zusammenarbeit.

Bericht: Ulrich Zechner

## Jahresbericht 2022 der Kommission Akademie Humangenetik

Prof. Dr. med. Christian Schaaf, Heidelberg (Sprecher)

Dr. rer. nat. Karl Hackmann, Dresden

Prof. Dr. med. Peter Krawitz, Bonn

Dr. rer. nat. Kerstin Ludwig, Bonn

Dr. med. Jan-Christoph Schöne-Bake, Hamburg (Junge Humangenetik)

Prof. Dr. med. Malte Spielmann, Kiel/Lübeck

Prof. Dr. Christian Thiel, Erlangen

Prof. Dr. med. Klaus Zerres, Aachen

Prof. Dr. med. Dr. rer. nat. Birgit Zirn, Stuttgart

**Kursorganisation**

GfH-Geschäftsstelle

Anja Rössler und Marie-Louisa Schiller

info@akademie-humangenetik.de

www.akademie-humangenetik.de

### Rückblick 2022

Im Jahr 2022 wurden 15 Kurse mit insgesamt 32 Kurstagen angeboten. Diese wurden von insgesamt 427 Teilnehmerinnen und Teilnehmer besucht, darunter 269 Kolleginnen und Kollegen aus anderen Fachgebieten. Die Kursevaluationen waren durchgehend positiv.

Auf Vorschlag der Kommission „Akademie Humangenetik“ wurden die Referentenhonorare für die Erstellung neuer Lehrinhalte und die Möglichkeit der Aufteilung des Leitungshonorars in die Tarif- und Honorarordnung 2023 aufgenommen und vom Vorstand der GfH in seiner Sitzung am 14.11.2022 verabschiedet.

### Ausblick 2023

Zum ersten Mal nach Beginn der Pandemie wird die Akademie Humangenetik im Jahr 2023 wieder Präsenzkurse anbieten. Aber auch weiterhin wird von den Möglichkeiten der Online-Kurse und ggf. Hybrid-Kurse Gebrauch gemacht werden. Neue Kursformate, wie interaktive, zweizeitige Kurse mit dazwischen geschalteten Übungsaufgaben, werden aufgrund der positiven Erfahrungen auch 2023 wieder Anwendung finden.

Ein neues Weiterbildungsformat im Bereich Zytogenetik und im Bereich Tumorzytogenetik wird erstmals im März 2023 stattfinden. Diese richten sich insbesondere an Kolleginnen und Kollegen, die sich in der Weiterbildung zum Fachhumangenetiker/Fachhumangenetikerin befinden.

Die Kommission freut sich auf ein gutes und vielfältiges Kursprogramm im Jahr 2023 mit vielen Möglichkeiten zum gemeinsamen Lernen, der Weiterbildung und des kollegialen Austauschs.

### Dank

An dieser Stelle sei allen Dozentinnen/Dozenten des vergangenen Jahres ein herzlicher Dank ausgesprochen!

Darüber hinaus gilt unser besonderer Dank Prof. Tiemo Grimm, Würzburg und Klaus Zerres, Aachen, die einen wesentlichen Anteil an der erfolgreichen Organisation und Durchführung der 72-Stunden-Kurse hatten.

Dank auch an die Geschäftsstelle der GfH, die das operative Management der Kurse übernimmt: Anja Rössler und Marie-Louisa Schiller stehen immer mit Rat und Tat zur Seite und sind für die schnelle Umsetzung und reibungslosen Abläufe der Kurse verantwortlich.

Bericht: Christian Schaaf

## Zeitschrift Medizinischegenetik – Jahresbericht 2022

**Schriftleiter:**

Prof. Dr. med. Markus Nöthen, Bonn (federführend)

Prof. Dr. med. Reiner Siebert, Ulm

Prof. Dr. med. Malte Spielmann, Lübeck

Prof. Dr. med. Dagmar Wieczorek, Düsseldorf

Prof. Dr. med. Johannes Zschocke, Innsbruck

**Wissenschaftlicher Beirat:**

Dr. med. Nuria Brämswig, Essen

Prof. Dr. rer. nat. Frank Kaiser, Lübeck

Prof. Dr. rer. nat. Eva Klopocki, Würzburg

Prof. Dr. med. Ingo Kurth, Aachen

Dr. med. Ilona Krey, Leipzig

Prof. Dr. med. Deborah Bartholdi, Bern (SGMG)

Prof. Dr. med. Michael Speicher, Graz (ÖGH)

### Themenschwerpunkte

Im Jahr 2022 wurden vier Ausgaben der Zeitschrift mit jeweils spezifischen Themenschwerpunkten veröffentlicht: 

**Table:** Themenschwerpunkte 2022

**Ausgaben**	**1-2022**	**2-2022**	**3-2022**	**4-2022**
**Schwerpunkte**	**Genetic Newborn Screening in Germany**	**Reduced Penetrance in Hereditary Movement Disorders**	**Epilepsy and Genetics**	**Functional Genomics Meets Human Genetics**
**Wissenschaftliche Koordination**	**Holger Tönnies, Uta Nennstiel**	**Christine Klein, Frank Kaiser**	**Johannes Lemke**	**Kerstin Ludwig,** **Malte Spielmann**

Die Inhalte der Zeitschrift für das Jahr 2023 wurden in einer gemeinsamen Videokonferenz mit Schriftleitern und wissenschaftlichem Beirat der Zeitschrift geplant. Folgende Themenschwerpunkte sind für die vier Ausgaben des Jahres 2023 vorgesehen:

**Table:** Themenschwerpunkte 2023

**Ausgaben**	**1-2023**	**2-2023**	**3-2023**	**4-2023**
**Schwerpunkte**	**Dermatogenetics**	**Genome Sequencing in Rare Disorders**	**Sex Diversity**	**Liquid Biopsies**
**Wissenschaftliche Koordination**	**Regina Betz,** **Ulrike Hüffmeier**	**Peter Krawitz, Tobias Haack**	**Olaf Hiort,** **Malte Spielmann**	**Michael Speicher,** **Ariana Hallermayr**

### Bereitstellung der Zeitschrift für die Mitglieder

Beim Versand der Zeitschrift gab es im Jahr 2022 seitens des Verlages leider einige Schwierigkeiten. Dies hatte eine verspätete Aussendung der medgen 02-2022 zur Folge.

Der Open Access Zugang zu den Übersichtsartikeln hat sich bewährt, was sich in den gestiegenen Zugriffszahlen widerspiegelt.

### Zitationsindices

Die positive Entwicklung der letzten Jahre spiegelt sich in der Steigerung verschiedener Zitationsindices wider („CiteScore“, „Journal Citation Indicator“, „Total Citations“). So stieg der „CiteScore“ von 0,3 in 2018 auf 2,6 in 2020 (!). Der „Impact Factor“ ist allerdings von 1.72 (2020) auf 0.84 (2019) gefallen. Bei diesem Index sind die Ausschläge wegen des kürzeren Betrachtungszeitraums erwartungsgemäß größer sowie zwischenzeitliche Änderungen in der Berechnungsweise zu berücksichtigen.

Dennoch weisen andere Indices, die keinen so kleinen, 2-jährig rollierenden Betrachtungszeitraum wie der IF haben, sondern eher kumulativ betrachten, eine ständig wachsende Menge an Zitaten von medgen-Artikeln auf.

Sowohl der „Journal Citation Indicator (JCI)“ als auch die „Total Citations“ weisen nach oben und belegen damit, dass sich die medgen in die richtige Richtung bewegt.

Die substantielle Steigerung des CiteScores weist die gleiche positive Entwicklung der Zitationen auf, von 0,3 in 2018 auf 2,6 in 2020 (!):

### TOP 3 Most Viewed Manuscripts

Die 2022 bei den Lesern beliebtesten Artikel des Jahres waren:

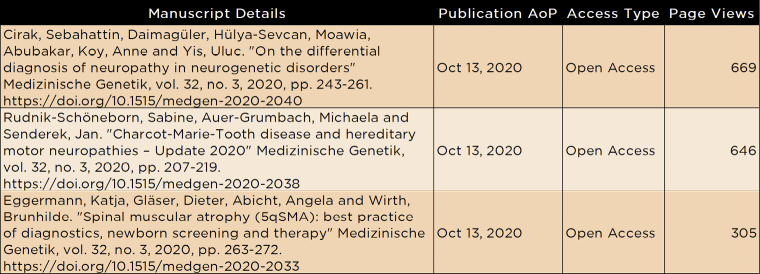



### PubMed-Listung

Die Bewerbung bei PubMed Central wird aktuell, dank der nun erreichten regelmäßigen Veröffentlichung der Zeitschrift, in Zusammenarbeit mit dem Degruyter Verlag erfolgen.

Hierfür sind weiterhin gemeinsame Anstrengungen bei der pünktlichen Abgabe der Manuskripte und der zeitnahen Begutachtung notwendig.

Bericht: Markus Nöthen

## Jahresbericht 2022 – Leitlinien-Kommission der GfH

Dr. med. Bernd Auber, MBA, Hannover (Sprecher)

Prof. Dr. med. Angela Abicht, München

Prof. Dr. med. Rami Abou Jamra, Leipzig

Dr. med. Diana Mitter, Düsseldorf

Dr. rer. nat. Katja Eggermann, Aachen

PD. Dr. med. Alma Küchler, Essen

Prof. Dr. med. Johannes Lemke, Leipzig

Dr. med. Verena Steinke-Lange, München

**BVDH-Delegierte**

Dr. rer. nat. Frank Oeffner, Neu-Ulm

Dr. rer. nat. Gabriele Wildhardt, Frankfurt/M.

### Benennung von MandatsträgerInnen durch die GfH – Entwicklung eines standardisierten Verfahrens

Die Zahl der bei der Arbeitsgemeinschaft der Wissenschaftlichen Medizinischen Fachgesellschaften (AWMF e. V.) angemeldeten Leitlinien (LL), bei denen eine humangenetische Expertise gefordert ist, steigt stetig an. Um ein hohes Maß an Beteiligung möglichst vieler GfH-Mitglieder sowie ein transparentes Vorgehen bei gleichzeitiger Machbarkeit im Alltag zu gewährleisten, hat die Kommission in Zusammenarbeit mit der GfH-Geschäftsstelle (GS) und dem GfH-Vorstand ein standardisiertes Verfahren zur Benennung von MandatsträgerInnen entwickelt. Es gibt zwei Möglichkeiten, wie die die Beteiligung der GfH an LL-Projekten anderer Fachgesellschaften realisiert werden kann.

1. Bei einer offiziellen Anfrage einer federführenden Fachgesellschaft an die GS wird folgendes Verfahren angewandt:

**Abbildung 1:**
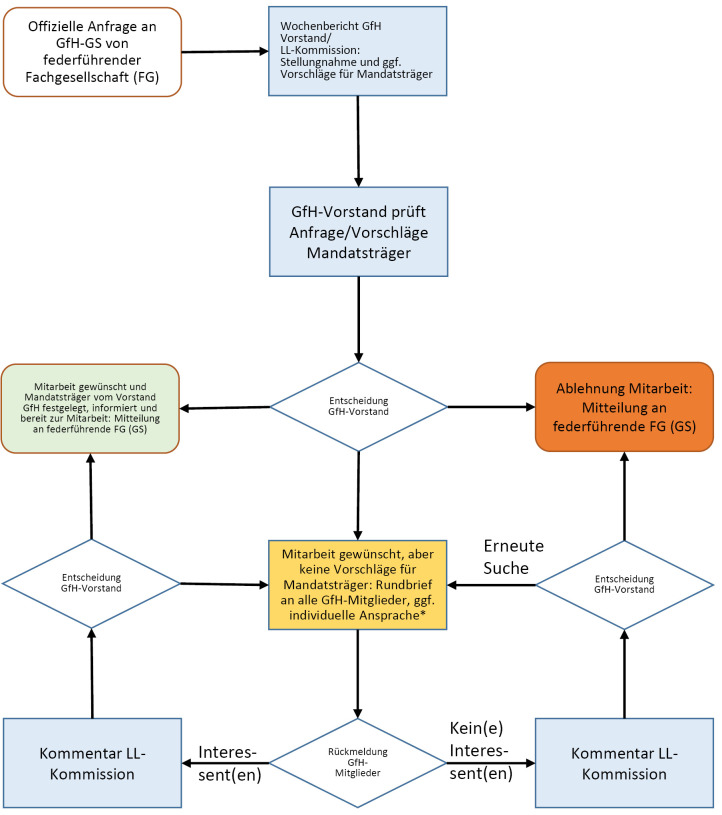
offizielle Anfrage einer federführenden Fachgesellschaft an die GfH-Geschäftsstelle (*Brief wird durch LL-Kommission formuliert und durch die GS versandt)

- Potentiell relevante LL in Entstehung/Überarbeitung/bei der AWMF angemeldet, bisher keine Beteiligung der GfH:

**Abbildung 2:**
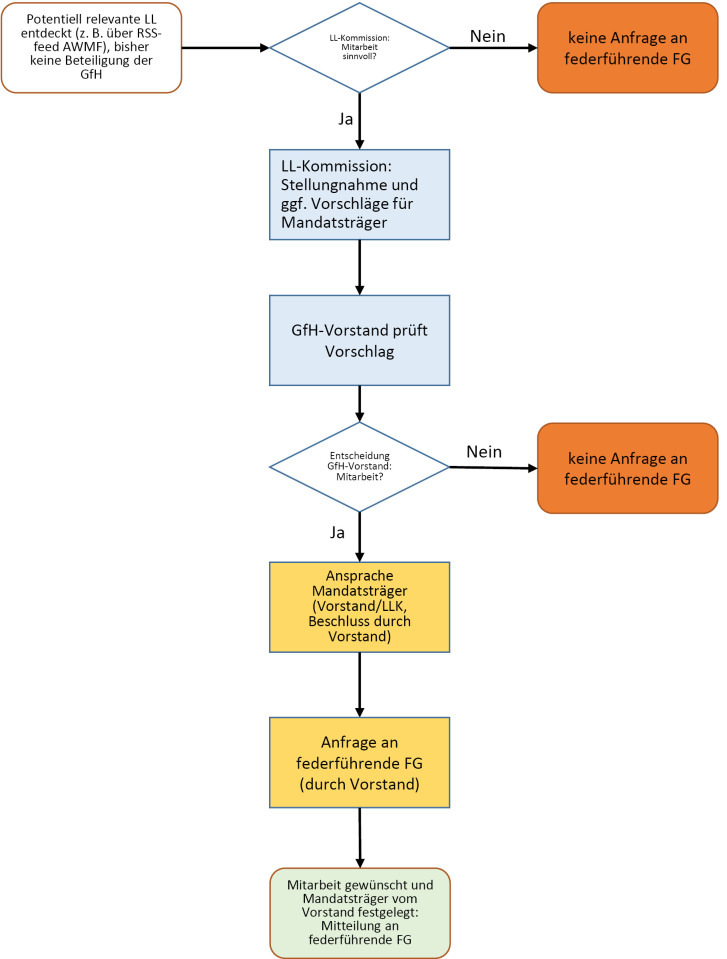
Potentiell relevante LL in Entstehung/Überarbeitung/bei der AWMF angemeldet

Beide Verfahren wurden nach Genehmigung durch den Vorstand in 2022 bereits vielfach erfolgreich angewendet. Die GfH strebt an, für jedes LL-Projekt zwei MandatsträgerInnen zu benennen – hauptamtlich und in Stellvertretung. Hierdurch soll zum einen die individuelle Arbeitslast reduziert werden und auch das Heranführen jüngerer GfH-Mitglieder an die LL-Arbeit ermöglicht werden. Es wird insbesondere angestrebt, Mitglieder der „Jungen Humangenetik“ für eine Mitarbeit zu gewinnen.

### Monitoring der auf der AWMF-website angemeldeten LL

Auch wenn eine LL humangenetische Expertise erfordert, sind in der Vergangenheit zahlreiche LL ohne (offizielle) Mitarbeit der GfH entstanden. Um bei möglichst vielen humangenetisch relevanten LL mitwirken zu können oder zumindest der federführenden Fachgesellschaft durch den Vorstand der GfH eine Mitarbeit anzubieten, hat die LL-Kommission das folgende Verfahren eingeführt: auf der Website der AWMF wird ein RSS Feed angeboten, in dem über den aktuellen Stand aller LL-Projekte berichtet wird (Neuanmeldung, Überarbeitung, Veröffentlichung). Dieser Feed wird von der LL-Kommission und der GS regelmäßig auf relevante Projekte durchgesehen. Wird eine potentiell relevantes LL identifiziert, wird das oben unter „2.“ beschriebene Verfahren angewendet.

### Mitarbeit an LL-Projekten und geplante LL unter Federführung der GfH

Die Mitglieder der GfH sind an zahlreichen LL-Projekten beteiligt. Ende 2022 waren bei der AWMF 30 in Entwicklung befindliche LL-Projekte bei denen MandatsträgerInnen der GfH mitwirken, angemeldet. Eine Auflistung dieser LL-Projekte kann auf der Website der AMWF unter diesem link eingesehen werden: https://register.awmf.org/de/suche#versionlabel=Registration&association=078&sorting=relevance

Der große Dank der LL-Kommission geht an alle Mitglieder der GfH, die an dieser Vielzahl an LL-Projekten teilgenommen haben bzw. die sich aktuell einbringen und so die Fahne der Humangenetik hochhalten. In Anbetracht der Tatsache, dass humangenetische Themen in immer mehr Bereiche der Medizin Einzug halten, ist dies für eine so kleine Fachgesellschaft wie die unsere eine große Herausforderung und nur durch den unermüdlichen Einsatz der GfH-Mitglieder zu bewältigen. In 2022 konnte für jedes als relevant identifizierte LL-Projekt eine Mandatsträgerin/ein Mandatsträger gewonnen werden!

Abschließend danke ich allen Kommissionsmitgliedern, dem Vorstand und der Geschäftsstelle der GfH für die hervorragende und verlässliche Zusammenarbeit.

Bericht: Bernd Auber

## Bericht 2022 der Programmkommission Tumorgenetische Arbeitstagung (TGA)

Dr. med. Lana Harder, Kiel (Sprecherin)

Dr. rer. nat. Sönke Arps, Hamburg

Prof. Dr. med. Gudrun Göhring, Hannover (Tagungspräsidentin 2022)

Prof. Dr. med. Detlef Haase, Göttingen

Prof. Dr. med. Gundula Kadgien, Berlin/Rostock (Tagungspräsidentin 2023)

Prof. Dr. rer. nat. Steffi Urbschat, Homburg Saar

Zu den Hauptaufgaben der TGA-Kommission gehört die Vorbereitung der jährlichen Tumorgenetischen Arbeitstagung**.** Die 34. TGA fand als Präsenz-Veranstaltung vom 12. bis 14.05.2022 statt und wurde vom Institut für Humangenetik der MHH (Tagungspräsidentin Prof. Dr. med. Gudrun Göhring) in enger Zusammenarbeit mit der GfH-Geschäftsführung organisiert. 121 Teilnehmer kamen in Barsinghausen bei Hannover zusammen, darunter 22 ÄrztInnen, 55 NaturwissenschaftlerInnen sowie 22 MTA/BTA/CTA. Durch 10 Industriestände waren die Industriesponsoren vor Ort vertreten.

Der erste Tag der TGA 2022 war den Vorträgern den Sponsoren, der Qualitätssicherung und dem Tagungschromosom 5 gewidmet. Das humangenetische Quiz, organisiert von Herrn Prof. Oskar Haas (ÖGH) war nicht nur lehrreicht, sondern hat alle Teilnehmer mehrfach zum Lachen gebracht.

Der zweite Tag der TGA 2022 startete mit einem Symposium der JAZZ Pharmaceuticals zum Thema „Neue Methoden der AML-Diagnostik“.

Der diesjährige Lore-Zech-Preis ging an Dr. rer. nat. Yvonne Lisa Behrens, MHH Hannover. Sie erhielt diese Auszeichnung für die Arbeit „A novel classification of hematologic conditions in patients with Fanconi anemia“ aus 2021, die sie als Erstautorin in *Haematologica* veröffentlicht hatte. Die Laudatio hielt Prof. Dr. Detlef Haase. Nach der Preisverleihung erfolgten der MTA-Workshop, die Besichtigung der Industrieausstellung sowie die Sitzung der TGA-Programmkommission.

Am Ende des MTA-Workshops 2021 haben die TeilnehmerInnen den Wunsch geäußert, diese Fortbildungsveranstaltung zukünftig nicht nur im Rahmen der TGA als Präsenzmeeting, sondern zusätzlich als zweite Online-Veranstaltung einmal jährlich fortzusetzen. Auch wenn die TeilnehmerInnen des Workshops eine intensive Diskussion bei der TGA 2022 hatten, haben sie sich gegen eine zweite Online-Veranstaltung in diesem Jahr entschieden.

Die nächste TGA 2023 wird von Prof. Gundula Kadgien organisiert und findet vom 20. bis 22.04.2023 in Rostock statt.

Ich bedanke mich bei allen Mitgliedern der Kommission und insbesondere bei Frau Rössler und Frau Schiller von der GfH-Geschäftsleitung für die sehr angenehme und produktive Zusammenarbeit.

Bericht: Lana Harder

## Bericht 2022 des Arbeitskreises Telemedizin

Theresa Neuhann, München (Sprecherin)

Dr. med. Johanna Tecklenburg, Hannover/Mainz (GfH)

Dr. med. Nils Rahner, Bonn (BVDH)

Dr. med. Nikola Dikow, Heidelberg (GfH)

Dr. med. Julia Schreml, Köln (GfH)

Dr. med. Dipl. biol. Martina Kreiß, Bonn (GfH)

Dr. med. Eva-Maria Prott, Wuppertal (BVDH)

Dr. med. Bernt Schulte, Hannover (BVDH)

Dr. med. Isolde Schreyer, Jena (BVDH)

Die Mitglieder des AK Telemedizin wurden sowohl von BVDH als auch GfH ernannt, somit ist er der einzige Arbeitskreis, der die humangenetischen Fachgesellschaften übergreifend agiert.

Am 28.03.2022 hat es erneut ein virtuelles Treffen gegeben. In diesem Rahmen wurden die aktuellen Anwendungen der Telemedizin in der Humangenetik – z. B. im Rahmen des Innovationsprojektes der MHH (Vorstellung durch Frau Dr. J. Tecklenburg) – vorgestellt, sowie künftige Anwendungs-, wie z. B. die Möglichkeit von Telekonsilen, und Kooperationsmöglichkeiten erörtert. Weiterhin fand eine lebhafte Diskussion zu den praktischen Erfahrungen und aktuellen Herausforderungen statt. Letzteres betrifft insbesondere – wenn auch nicht nur – die Abrechnungsmodalitäten.

Im Rahmen des virtuellen Treffens wurde beschlossen, gemeinsam zum Stellenwert der Telemedizin in der Humangenetik, der Anwendungsbereiche sowie der anzustrebenden Voraussetzungen (z. B. regionaler Bezug, Kombination telemedizinische Sprechstunde und persönliche Vorstellung) ein Positionspapier zu veröffentlichen, das GfH und BVDH im September 2022 zur Verfügung gestellt wurde. Dies wurde im Rahmen der BVDH-Jahrestagung am 25.11.2022 durch Frau Dr. Neuhann vorgestellt.

Aktuell findet eine gemeinsame Abstimmung mit der Qualitätskommission (QK) „Genetische Beratung und Klinische Genetik“ des BVDH statt, um gemeinsam eine AK-QK-übergreifende Stellungnahme zur telemedizinischen Patientenbetreuung in der Humangenetik zu erwirken.

Bericht: Teresa Neuhann

## Bericht 2022 des Arbeitskreises Präimplantationsdiagnostik (AK-PID)

Dr. rer. nat. Udo Koehler, München (Sprecher)

Dr. med. Teresa Neuhann, München (Sprecherin)

Prof. Dr. med. Isabel Diebold, München

Dr. rer. nat. Daniel Findeis, Freiburg

Dr. rer. nat. Päivi Forsblom, München

Dr. med. Christof Hammans, Dortmund

Dr. rer. nat. Andreas Hehr, Regensburg

Prof. Dr. med. Ute Hehr, Regensburg

Dr. rer. nat. Katrin Hinderhofer, Heidelberg

Dr. med. Laura Holthöfer, Mainz

Dr. rer. nat. Matthias Linke, Mainz

Dr. med. Moritz Meins, Hannover

Dr. med. Claudia Nevinny-Stickel-Hinzpeter, München

Dr. rer. nat. Katarzyna Osetek-Müller, Martinsried

Dr. med. Lisa Peterson, Martinsried

Dr. med. Eva Schwaibold, Heidelberg

Dr. med. Malte Spielmann, Lübeck

Dr. rer. nat. Alexandra Tibelius, Heidelberg

Dr. rer. nat. Sarah Volpert, Dortmund

Dr. rer. nat. Annett Wagner, Martinsried

Dr. med. Eva Wohlleber, Freiburg

Dr. rer. nat. Susanne Zahn, Hannover

Prof. Dr. med. Christine Zühlke, Lübeck

Das Jahrestreffen des Arbeitskreises Präimplantationsdiagnostik (AK-PID) fand am 09.12.2022 als virtuelles Zoom-Meeting statt. Jedes der zehn deutschen PID-Zentren war vertreten.

Über acht Jahre nach der Zulassung des ersten PID-Zentrums ist die Präimplantationsdiagnostik fester Bestandteil der humangenetischen Diagnostik. Zwei Untersuchungsverfahren an Trophoblasten nach Trophektodermbiopsie werden von der Mehrheit der deutschen PID-Zentren angewendet: (1) eine SNP-Microarray basierte Methode für die PID monogener Erkrankungen (PGT-M) und (2) ein auf Sequenziertechnologie basierter Ansatz (*shallow sequencing*) für die PID struktureller (PGT-SR) und numerischer (PGT-A) Chromosomenveränderungen. Eine Polkörperdiagnostik wird in den PID-Zentren nur noch in Ausnahmefällen durchgeführt.

### Ethikkommissionen

Problematisch hat sich die Situation der Bayerischen Ethikkommission entwickelt. Die von ihr zu beratenden Fälle hatten einen Anteil von 78 % aller 496 im Jahr 2021 in Deutschland gestellten Ethikanträge für Präimplantationsdiagnostik, ein Zuwachs von 9 % im Vergleich zum Jahr 2020 mit 372 Anträgen. Mit 386 zu beratenden Fällen im Jahr 2021 ist die Kapazitätsgrenze der Bayerischen Ethikkommission von 300 Anträgen pro Jahr überschritten. Dies führt zu langen Wartezeiten für die Antragsstellerinnen von bis zu einem halben Jahr. Abhilfe könnte die Verteilung der Ethikanträge auf alle 4 Ethikkommissionen in Deutschland schaffen, neben der Bayerischen Ethikkommission mit Sitz in München sind dies die Kommissionen in Hamburg, Stuttgart und Düsseldorf. Der Antrag könnte bei einer für den Wohnsitz der Antragstellerin zuständigen Ethikkommission gestellt werden anstatt bei der Ethikkommission, in deren Bundesland/Verbund von Bundesländern das PID-Zentrum ansässig ist, das die Untersuchung durchführt. Voraussetzung hierfür wäre (1) eine Änderung der in den die einzelnen Bundesländern gültigen und gesetzlich geregelten Verordnungen für PID (PIDV) und vor allem (2) eine Angleichung der sehr unterschiedlichen Gebührensätze, die im Durchschnitt in Stuttgart, Düsseldorf und Hamburg das Zehnfache der Gebühren der Bayerischen Ethikkommission betragen. Die mögliche Umsetzung dieses Vorhabens kann sicher nicht kurzfristig erfolgen, so dass die Antragsstellerinnen in Bayern weiterhin mit langen Wartezeiten rechnen müssen.

### Zulassungsmodalitäten

Ein weiterer Diskussionspunkt des Jahrestreffens waren die unterschiedlichen Zulassungsmodalitäten für PID-Zentren und den mit Ihnen kooperierenden reproduktionsmedizinischen Zentren in den einzelnen Bundesländern. Während in Bayern vier PID-Zentren zugelassen sind, die mit mehreren reproduktionsmedizinischen Zentren länderübergreifend kooperieren, sind andere Bundesländer zum Teil nur durch ein einzelnes PID-Zentrum vertreten (Bsp. Nordrhein-Westphalen) oder die Kooperation des PID-Zentrums ist nur auf ein reproduktionsmedizinisches Zentrum beschränkt (Bsp. Niedersachsen). Eine Angleichung der Zulassungsmodalitäten kann dazu beitragen, dass die PID-Untersuchungen gleichmäßiger auf alle Zentren verteilt werden können. Derzeit werden in den vier bayerischen PID-Zentren zwei Drittel aller in Deutschland beantragten PIDs durchgeführt.

### Aneuploidieuntersuchung bei monogener Präimplantationsdiagnostik (PGT-M)

Chromosomenaberrationen können nicht nur Folge einer balancierten Chromosomenveränderung beim Paar sein, sondern auch sporadisch in jedem Embryo vorliegen. Einige Methoden, die im Rahmen einer PID für monogene Erkrankung Anwendung finden, ermöglichen die gleichzeitige Untersuchung numerischer Chromosomenanomalien (Aneuploidieuntersuchung). Zu diesen Methoden gehört zum Beispiel ein SNP-Microarray basiertes Verfahren, das mehrere PID-Zentren durchführen. Eine den Embryo belastende Doppelbiopsie muss dabei nicht erfolgen. Voraussetzung ist eine ausführliche humangenetische Beratung, die das Paar über mögliche zu erwartende Zusatzbefunde und die diagnostischen Grenzen aufklärt. Zudem muss das Vorhaben, eine Aneuploidieuntersuchung neben der PID für die monogene Erkrankung durchführen zu lassen, im Antrag auf Durchführung einer PID deutlich gekennzeichnet sein. Nur wenn die Ethikkommission dieser zusätzlichen Untersuchung zustimmt, darf dieses Ergebnis auch mitgeteilt werden.

### Mosaike und segmentale Aneuploidien

Weiteres Thema der Sitzung war das Vorgehen bei Mosaikbefunden und segmentalen Aneuploidien de novo. Chromosomale Mosaike werden in etwa 5–10 % der untersuchten Proben nachgewiesen, etwas seltener sind segmentale Aneuploidien, die *de novo* aufgetreten sind und nicht Folge der beim einem der Partner vorliegenden strukturellen Chromosomenveränderung sind. Die *Preimplantation Genetic Diagnosis International Society* (*PGDIS*) [1] und die *European Society of Human Reproduction and Embryology* (*ESHRE*) [2] geben Empfehlungen zum Vorgehen bei Vorliegen eines Mosaikbefundes. Es besteht Konsens der deutschen PID-Zentren, diesen Empfehlungen zu folgen. Ebenfalls einheitlich wird die Befundung von Ergebnissen für eine PID bei Disposition für strukturelle Chromosomenveränderungen gehandhabt, wenn die Größe eines der zu untersuchenden Chromosomensegmente unterhalb der methodischen Auflösungsgrenze von etwa 5 Megabasen liegt. In diesem Fall muss dies im Befund ausdrücklich als Ergebnis mit eingeschränkter Aussagekraft dokumentiert werden.

Alle oben genannten Konstellationen erfordern die besondere Notwendigkeit einer humangenetischen Beratung vor und nach einer PID.

### § 20c AMG und Datenübermittlung an das Paul-Ehrlich-Institut

Klarheit besteht seit Herbst 2022 darüber, dass die humangenetische Einrichtung, die die PID durchführt, keiner Erlaubnis nach § 20 AMG (Arzneimittelgesetz) bedarf und auch nicht in die Erlaubnis nach § 20c der kooperierenden reproduktionsmedizinischen Zentren eingebunden werden muss. Die Tabellen zur Erfassung der von den PID-Zentren durchgeführten PID-Zyklen wurden vom Paul-Ehrlich-Institut (PEI) überarbeitet und haben in der nun bestehenden Form bis auf Weiteres Bestand.

### Datenübermittlung an die ESHRE und das DIR

Die Datenübermittlung der in den PID-Zentren durchgeführten Zyklen an das ESRE-PGT-Consortium (https://www.eshre.eu/Data-collection-and-research/Consortia/PGD-Consortium) wird uneinheitlich gehandhabt. Nur wenige Zentren übermitteln ihre Daten direkt an das PGT-Consortium. Nach wie vor ist nicht geregelt, wer die Daten übermittelt, was insbesondere der Tatsache geschuldet ist, dass einige PID-Zentren mit zahlreichen reproduktionsmedizinischen Einrichtungen kooperieren, die ihrerseits Daten selbst übermitteln, eine doppelte Übermittlung muss vermieden werden. Geplant ist, das im Deutschen IVF-Register, DIR (https://www.deutsches-ivf-register.de) gebotene Modul zu nutzen. Es wird eine Herausforderung für die PID-Zentren bleiben, genaue Angaben zum Follow-up einer PID zu erhalten, da bislang keine Datenbank existiert, die einheitlich für alle Zentren dokumentiert, welcher Embryo transferiert wurde und zu welchem Ergebnis (Implantation/Schwangerschaft/Geburt/Fehlgeburt) dieser Transfer führte.

### Neue Methoden und In-vitro Diagnostik-Richtlinie der EU (IVDR)

Einige Produkte für die genetischen Untersuchungen im Rahmen der PID werden 2023 durch neue Versionen oder neue Formate ersetzt. Zum Zeitpunkt der Sitzung der AK-PID konnten diesbezüglich noch keine Zeitangaben gemacht werden. Eine weitere Herausforderung wird die Umsetzung der EU-Verordnung für In-vitro-Diagnostica (IVDR) [3] sein, die seit 26.05.2022 verpflichtend anzuwenden ist. Zu hoffen ist, dass die neuen Produkte der Hersteller den Vorgaben der IVDR entsprechen, um eine den Richtlinien entsprechende Diagnostik durchführen zu können.

### Nicht-invasive PID

Eine Nicht-Invasive PID ist die genetische Untersuchung des Kulturmediums der Blastozystenkultur, seltener auch der Blastocoelflüssigkeit. Sie gewinnt international zunehmend an Bedeutung, um neben den morphologischen auch genetische Faktoren zur Bestimmung der Transferempfehlung eines Embryos vornehmen zu können. In erster Linie bezieht sich diese Untersuchung auf die PGT-A, also die PID für numerische Chromosomenaberrationen. Es soll gewährleistet werden, dass nur euploide, chromosomal unauffällige Embryonen für einen Transfer berücksichtigt werden [4] [5]. Nach aktueller Sichtweise handelt es sich bei der DNA, die aus dem Blastozystenkulturmedium gewonnen wird, um zellfreie DNA, also nicht um *Zellen eines Embryos*, die dem § 3a ESchG unterliegen würden. Zum jetzigen Zeitpunkt ist weder dies belegt noch zeigen die Untersuchungsergebnisse, dass die Methode sensitiv und spezifisch genug ist, eine PID nach Trophektodermbiopsie zu ersetzen. Es besteht Konsens, dass noch weitere Studien zur Nicht-Invasiven PID erforderlich sind und sie von deutschen PID-Zentren nicht angewendet werden sollte.

### References

[1]LeighDCramD SRechitskySHandysideAWellsDMunneSKahramanSGrifoJKatz-JaffeMRubioCViottiMFormanEXuKGordonTMadjunkovaSQiaoJChenZ-JHartonGGianaroliLSimonCScottRSimpsonJ LKulievAPGDIS position statement on the transfer of mosaic embryos 2021Repromed Biomed Online. 2022 Jul;45(1)1925PMID: 3552370710.1016/j.rbmo.2022.03.01335523707[2]ESHRE Working Group on Chromosomal MosaicismESHRE survey results and good practice recommendations on managing chromosomal mosaicismHum Reprod Open. 2022 Nov 7;2022(4), PMID 3634914410.1093/hropen/hoac044PMC963742536349144[3]Verordnung (EU) 2017/46 des Europäischen Parlaments und des Rates vom 05.04.2017 über In-Vitro-Diagnostica und zur Aufhebung der Richtlinie 98/79/EG und des Beschlusses 2010/227/EU der KommissionVerordnung (EU) 2017/46 des Europäischen Parlaments und des Rates vom 05.04.2017 über In-Vitro-Diagnostica und zur Aufhebung der Richtlinie 98/79/EG und des Beschlusses 2010/227/EU der Kommission[4]RogersAMenezesMKaneS CZander-FoxDHardyTPreimplantation Genetic Testing for Monogenic Conditions: Is Cell-Free DNA Testing the Next Step? Mol Diagn Ther2021 Nov;25(6)683690PMID 3449548310.1007/s40291-021-00556-034495483[5]Navarro-SánchezLGarcía-PascualCRubioCSimónCNon-invasive preimplantation genetic testing for aneuploidies: an updateReprod Biomed Online 2022 May;44(5)817828PMID 3530729810.1016/j.rbmo.2022.01.01235307298

Bericht: Udo Koehler

## Bericht 2022 des GfH-Delegierten über die Arbeit im Sektorkomitee Medizinische Laboratorien der Deutschen Akkreditierungsstelle (DAkkS)

In der November-Sitzung 2022 des Sektorkomitees Medizinische Laboratorien der DAkkS wurde über die überarbeitete neue Version der DIN EN ISO 15189:2022-12 Medizinische Laboratorien – Anforderungen an die Qualität und Kompetenz berichtet, die in der englischen Ausgabe „Medical laboratories – Requirements for quality and competence“ bereits erschienen ist. Die deutschsprachige Ausgabe wird für 2023 erwartet. Die überarbeitete Version zeigt die ausgeprägte Annäherung der ISO 15189 an die ISO/IEC 17025 für Prüf- und Kalibrierlaboratorien, die bereits früher überarbeitet worden war. Außerdem wurden die Aspekte der Akkreditierung im Bereich POCT, bisher gemäß ISO 22870, mit in die Norm einbezogen. Die ISO 15189 präsentiert sich deutlich ergebnisorientierter und mit einem Schwerpunkt auf dem Risikomanagement. Gestärkt wurde der Aspekt der Sicherstellung der Kompetenz des Personals. Verschiedene Teile wurden vereinfacht und eindeutiger gefasst. Durch die Angleichung der Inhalte wird zukünftig die Erfüllung mehrerer Normen erleichtert. Die Neuordnung der Normpunkte führte zu einem Tausch der Positionen des Managementsystems (nun am Ende) mit den Anforderungen an die personellen und technischen Laboraspekte, die nun zu Beginn aufgeführt werden. Nach Inkrafttreten der neuen deutschsprachigen Version wird es eine Übergangsperiode bis zu deren verpflichtenden Verwendung bei Akkreditierungsverfahren geben. Diese Übergangsperiode beträgt üblicherweise drei Jahre, wurde aber noch nicht bekanntgegeben.

Ein weiterer Bericht betraf den Stand der Überarbeitung der Rili-BÄK. Der für die Humangenetik relevante Anhang B5 „Molekulargenetische und zytogenetische laboratoriumsmedizinische Untersuchungen“ ist seit zwei Jahren in intensiver Bearbeitung und wird voraussichtlich im ersten Quartal 2023 abgeschlossen werden.

Im Rahmen der Anfrage eines Laboratoriums zur Auflistung von Genen bei Panel-Diagnostik wurde auf den aktuellen Stand der Überarbeitung der Vorgaben der Humangenetik zur Darstellung der Untersuchungsverfahren des DAkkS-Formblattes FO-Antrag GB_ML-Diagnostik molekulare Humangenetik verwiesen. Es werden dort Aussagen getroffen zur gerichteten NGS-Panel-Analyse (kommerzielle und in-house Panel) bei benannten klinischen Verdachtsdiagnosen sowie zur NGS-basierenden Analyse bei ungerichteter klinischer Indikation (WES, WGS, NIPT). Diese Informationen werden Anfang 2023 auf der DAkkS Website erhältlich sein. In Hinblick auf die Benennung von Genen in Panels ist festzuhalten, dass für den Anhang der Akkreditierungsurkunde der DAkkS die Benennung sich bei einem Flexiblen Geltungsbereich der Akkreditierung auf Core-Gene pro Panel beschränken kann, die gesamten Gene aber in der von den Laboratorien zu führenden tagesaktuellen Liste der Untersuchungsverfahren benannt und dokumentiert werden müssen. Ein Verweis auf eine Website ist nicht möglich.

**Bericht:** Konstantin Miller

## Bericht 2022 der GfH-Delegierten über die Arbeit im Lenkungsgremium des FARKOR – Projektes zur Darmkrebsprävention bei familiärem Risiko

Das vom Innovationsfond geförderte Projekt FARKOR (Vorsorge bei **fa**miliärem **R**isiko für das **ko**lo**r**ektale Karzinom) wurde 2018 von der Felix-Burda-Stiftung initiiert und 2021 beendet; die Ergebnisse wurden 2022 veröffentlicht. Das wesentliche Ziel des Projektes war es festzustellen, welchen Nutzen eine frühere Darmkrebsfrüherkennung bei jungen Menschen mit Darmkrebserkrankungen in der Familie hat. Während bei Patient:innen über 50 Jahren die Zahl der Darmkrebserkrankungen infolge der Früherkennungs-Programme zurückgegangen ist, ist sie bei den unter 50-jährigen in den letzten Jahren gestiegen. Letztere werden durch die aktuell ab 50 Jahren empfohlene Früherkennung nicht erfasst und häufig erst spät diagnostiziert.

Im Rahmen dieses bayernweiten Modellprojektes sollten Risikopersonen für Darmkrebs bei den unter 50-jährigen identifiziert und eine einmalige frühzeitige Vorsorgeuntersuchung (Koloskopie oder immunologischer Stuhltest) angeboten werden. Nahezu alle bayerischen Krankenkassen nahmen an dem Projekt teil, die Knappschaft ist 2020 aus dem Projekt ausgeschieden. Die Konsortialführerschaft lag bei der Kassenärztlichen Vereinigung Bayerns (KVB). Das Lenkungsgremium des Projektes, das aus Vertretern verschiedener Fachgesellschaften (unter anderem der GfH) bestand, traf sich vierteljährlich mit den Konsortialpartnern, um über den Fortgang des Projektes und aktuell anstehende Aufgaben zu beraten.

Die Patientenrekrutierung durch über 500 bayerische Ärzt:innen (insbesondere Hausärzt:innen und Gastroenterolog:innen, aber auch Humangenetiker:innen und Ärzt:innen anderer Fachrichtungen) erfolgte von Oktober 2018 bis März 2021. Die entsprechenden Leistungen (einschließlich der Dokumentation), die nicht zum Regelleistungskatalog zählen, wurden den beteiligten Ärzt:innen gesondert vergütet.

Die Auswertung der Daten zeigte, dass 5.769 der 25.848 Befragten (22,3 %) eine positive Familienanamnese hinsichtlich Darmkrebs angaben (Patient:innen mit Hinweisen auf eine monogen erbliche Tumorprädisposition wurden von der Studie ausgenommen). Von diesen Patient:innen entschieden sich 2.783 daraufhin für eine Vorsorgeuntersuchung, etwas mehr als die Hälfte (ca. 57 %) mittels Koloskopie, die anderen über einen immunologischen Stuhltest (iFOBT). Über diese Untersuchungen wurden bei 287 Studienteilnehmer:innen (10 %) kolorektale Adenome bei einem mittleren Alter von 41,2 Jahren identifiziert. Bei 76 Patienten (2,7 %) lagen fortgeschrittene Adenome vor, bei vier Patient:innen (0,14 %) bereits eine Darmkrebserkrankung.

Die Adenomdetektionsrate bei den unter 50-jährigen mit positiver Familienanamnese war somit vergleichbar mit der, die für die Früherkennungskoloskopien in der Normalbevölkerung ab 50 Jahren angegeben wird. Dies deutet darauf hin, dass diese Patient:innen von einer früheren Darmkrebsvorsorge profitieren können. Es wird deshalb eine Früherkennung für diese Patient:innen mittels Koloskopie (alle 10 Jahre) bzw. iFOBT (alle 2 Jahre) im Alter von 30 bis 70 Jahren vorgeschlagen. Nach den Daten der FARKOR-Studie kann dieses Modell kosteneffektiv sein und erscheint im Hinblick auf vermiedene Todesfälle durch Darmkrebserkrankungen sinnvoll.

Bericht: Verena Steinke-Lange
